# PD-1 inhibitors provide new opportunities in conversion therapy for stage IV gastric cancer

**DOI:** 10.20892/j.issn.2095-3941.2022.0244

**Published:** 2022-06-09

**Authors:** Han Liang

**Affiliations:** 1 Gastric Cancer Center, Tianjin Medical University Cancer Institute & Hospital, National Clinical Research Center for Cancer, Key Laboratory of Cancer Prevention and Therapy, Tianjin, Tianjin’s Clinical Research Center for Cancer, Tianjin 300060, China

Gastric cancer is one of the most common malignant tumors worldwide. China is a large country in which gastric cancer ranks among the top 3 malignant tumors in terms of incidence, and related morbidity and mortality^1^. In the past 20 years, China has made the most outstanding achievements in the diagnosis and treatment of gastric cancer among countries worldwide. The overall 5-year survival rate of patients in China has increased by nearly 10%^2^. However, early gastric cancer accounts for only 20% of clinically confirmed cases, the overall effects of therapies for gastric cancer still must be improved^3^. The REGATTA study has confirmed that palliative surgery cannot improve the long-term survival of patients with stage IV gastric cancer^4^. To improve patient survival, multidisciplinary treatment has recently received substantial attention. Gastrointestinal surgeons in Japan have administered systemic chemotherapy to some patients with stage IV gastric cancer and performed R0 gastrectomy on some patients with down-staging disease, to potentially achieve long-term survival. Conversion therapy for gastric cancer is defined as a surgical treatment aimed at R0 resection after chemotherapy for tumors that are technically and/or oncologically unresectable, or marginally resectable. Yoshida et al.^5^ have proposed classification categories for stage IV gastric cancer according to the biology of the tumor burden. According to this classification, categories 2, 3, and 4 have been defined as marginally resectable or unresectable metastasis, macroscopic peritoneal dissemination, and peritoneal disease and other organ metastasis, respectively.

## Systemic chemotherapy

Kinoshita et al.^6^ have adopted a DCS regimen in conversion therapy for unresectable gastric cancer. In their study, 34 of 57 patients underwent surgery. The 3-year overall survival (OS) rate and median survival time (MST) of patients undergoing surgery were better than those of patients in the simple chemotherapy group. In the AIO-FLOT3 phase II study^7^, a FLOT regimen was used to treat gastric cancer; patients in group B were classified as having Yoshida category 2, 3, and partial 4 gastric cancer. The objective response rate (ORR) of patients in group B was 60% (36/60), and the R0 resection rate was 80.6% (29/36). The median OS in patients in this group was 22.9 months, and patients who proceeded to surgery had higher median OS than patients who were unable to undergo surgery and received FLOT alone (31 months *vs.* 16 months). This study indicated the patient survival benefits of conversion therapy and established paclitaxel as a first-line treatment for advanced gastric cancer.

For stage IV gastric cancer with peritoneal metastasis, medical oncologists from Japan have used combination intraperitoneal and systemic chemotherapy (ip and iv) and achieved remarkable clinical results. The subgroup analysis of the PHOENIX-GC study^8^ has suggested that intraperitoneal chemotherapy with paclitaxel can be used as a treatment mode for patients with moderate or greater abundance of ascites. Although it is not directly associated with the conversion therapy of stage IV gastric cancer with peritoneal metastasis, ip/iv chemotherapy provides the possibility of conversion therapy of stage IV gastric cancer with peritoneal metastasis, in a milestone in the treatment of peritoneal metastasis of gastric cancer, as demonstrated by the PHOENIX-GC study.

The CONVO-GC-1 study^9^, a multi-institutional, large-scale international retrospective cohort study on conversion therapy for stage IV gastric cancer conducted in Japan, Korea, and China, has also reported favorable outcomes for 1,206 patients with metastatic gastric cancer who underwent chemotherapy followed by attempted R0 resection for all disease. The MST for all resected patients was 36.7 mo (M) and those for R0, R1, and R2 resection were 56.6 M, 25.8 M and 21.7 M, respectively. Moreover, the MST for R0 patients was 47.8 M, 116.7 M, and 44.8 M in categories 1, 2, and 3, respectively, and was not reached in category 4. Conversion therapy for stage IV gastric cancer is safe and may serve as a new therapeutic strategy to improve patient survival, particularly with R0 resection.

## Hyperthermic interperitoneal chemotherapy (HIPEC)

Our previous study has confirmed that, compared with surgery alone, D2 plus HIPEC significantly increases the 5-year OS of patients with stage IIIb and different Borrmann types of gastric cancer^10^. The PILGRIM HIPEC-01 trial (NCT02356276) is currently ongoing in China to test the hypothesis that adjuvant HIPEC may decrease peritoneal recurrence rates in patients with high-risk resectable gastric cancers after radical gastrectomy. The patient recruitment has been completed, and a preliminary report has indicated a favorable safety profile in the HIPEC group^11^. The 3-year follow-up data are expected to be released by the end of this year. We are conducting the HIPEC-02 study (NCT05228743), in which the PHOENIX-GC trial regimen is used as the control treatment. For patients with stage IV gastric cancer with peritoneal metastasis, the treatment group is treated with paclitaxel ip/iv chemotherapy plus HIPEC. The aim of this study is to explore the role of HIPEC in the conversion therapy of stage IV gastric cancer with peritoneal metastasis.

## Chemotherapy combined with targeted drug therapy

The ToGA study^12^ has confirmed that trastuzumab in combination with chemotherapy can be considered a new standard option for patients with HER2-positive advanced gastric or gastro-oesophageal junction cancer (GC/GEJC). A small phase II study^13^ using trastuzumab combined with DCS regimen chemotherapy in the conversion therapy of 16 HER2-positive patients with unresectable metastatic gastric cancer has reported an ORR of 93.8% (15/16) and an R0 resection rate of 56.3% (9/16). The Ahead-G325 study^14^ has used apatinib (anti-angiogenesis antibody) combined with paclitaxel/S-1 in the conversion therapy of unresectable stage IV gastric cancer, and has reported an ORR and disease control rate (DCR) of 73.3% (22/30) and 93.3% (28/30). The R0 resection rate of patients who underwent surgery was 94.4% (17/18), and no serious surgery-associated complications were observed.

In our recent retrospective study^15^, 68 patients with unresectable stage IV gastric cancer received conversion therapy. 34 patients with peritoneal or/and ovarian metastasis received S1/paclitaxel (PTX)/apatinib (S1 60 mg, bid, d1–14; PTX 50 mg/m^2^ iv, d1 and 8; PTX 20 mg/m^2^ ip, d1 and 8; q3w; and apatinib 500 mg po, qd). A total of 34 patients with other non-curable factors were administered a regimen of SOX plus apatinib (oxaliplatin 130 mg/m^2^; S1 60 mg, bid, d1–14; and apatinib 500 mg, po, qd). After at least 3 cycles (3–9) of chemotherapy, 42 patients achieved partial response (PR), and 7 patients achieved stable disease, thus resulting in an ORR of 72.1%. A total of 46 patients received surgical treatment after conversion therapy, and the R0 resection rate was 93.5%. Patients who underwent surgery had a 1-year OS rate of 97.8%; for patients who did not undergo surgery, the 1-year OS was 54.5%, *P* < 0.001. As a conversion therapy, two-drug chemotherapy combined with apatinib provided a high ORR and R0 resection rate for unresectable stage IV gastric cancer, with favorable short-term effects.

## New exploration of the four-drug model in the era of immunotherapy

The extended analysis of data from the CheckMate649 study follow up for 24 months was reported at the ASCO GI annual meeting in 2022^16^. Nivolumab plus chemotherapy resulted in significant improvements in OS (*P* < 0.0001) and PFS (*P* < 0.0001) *vs.* chemotherapy alone in patients with a PD-L1 CPS 5 or more. The risk of death or disease progression decreased by 25%. In the subgroup analysis of Chinese participants^17^, for patients with CPS of 5 or more and CPS of 1 or more, the OS was 15.5 months and 14.3 months; the risk of death decreased by 46% and 39%; and the PFS was 8.5 months and 8.3 months. Nivolumab plus chemotherapy provides a new standard first-line treatment for advanced GC/GEJC. The initial findings of our single-arm phase II trial^18^ of perioperative sintilimab (PD-1 inhibitor) in combination with SOX for resectable locally advanced GC/GEJC were reported at the ASCO GI meeting in 2022. As of June 2021, 21 patients were enrolled, and all 21 patients had completed gastrectomy. Seven patients (33.3%) achieved pathological complete response (ypCR), 8 patients (38.1%) achieved major response (MPR, TRG 0–1), and all patients achieved R0 resection. No severe complications or deaths were associated with the operation. Adding sintilimab to the chemotherapy was encouraging as a perioperative treatment for resectable locally advanced GC/EGJC, on the basis of ypCR and MPR, and the safety was manageable.

The results of the protocol-specified first interim analysis of KEYNOTE-811^19^ have shown that adding pembrolizumab to standard therapy with trastuzumab and chemotherapy results in a statistically significant, clinically meaningful improvement in ORR, as compared with trastuzumab and chemotherapy alone, as a first-line therapy for unresectable or metastatic, HER2-positive GC/GEJC. The 74.4% ORR and 11% clinical complete remission observed with the addition of permbrolizumab to trastuzumab and chemotherapy represented a 22.7% and 8% improvement *vs.* transtuzmab and chemotherapy. Chinese researchers have reported the results from a phase II prospective study of preoperative SHR1210 (PD-1 inhibitor) in combination with trastuzumab and CapOX chemotherapy for HER2-positive locally advanced GC/GEJC: of the 22 patients, 4 did not complete neoadjuvant therapy, 2 refused surgery, and 16 underwent D2 gastrectomy. The ORR was 81%, and the R0 resection rate was 100%. Five patients achieved ypCR (31.3%), and a total of 9 patients achieved MPR (56.3%). This study successfully used neoadjuvant therapy with the four-drug model of KEYNOTE-811 for locally advanced gastric cancer.

On the basis of experience in the use of apatinib combined with chemotherapy in conversion therapy for stage IV gastric cancer, we designed the CO-STAR trial^20^ for HER2-negative unresectable gastric cancer, on the basis of KEYNOTE-811. Patients with peritoneal or/and ovarian metastasis received sintilimab 200 mg, iv, d1; Nab-PTX 200 mg/m^2^ iv, 3 h, d1; Nab-PTX 60 mg/m^2^, ip, d1; S1 60 mg, po, bid, d1–14; and apatinib 250 mg, po, qd, q3w. Patients with other non-curable factors were administered the same regimen without Nab-PTX 60 mg/m^2^, ip, d1, q3w. As of August 2021, 56 patients were enrolled. The median follow-up time was 5.0 months (range 0.7–16.3), Twenty-two (39.3%) patients had ≥2 unresectable lesions, and the most common unresectable lesions were No.16 lymph node (33, 58.9%), liver (10, 17.9%), peritoneum (21, 37.5%), and Krukenberg (2, 3.6%). Among 47 evaluable patients, 29 reached PR, and 17 reached stable disease. The ORR was 61.7%, and the DCR was 97.9%. After conversion therapy, 29 patients achieved surgical conversion, and R0 resection was completed in 28 patients. Five patients (17.2%) achieved ypCR (TGR 0), and 7 patients (24.1%) achieved MPR (TRG 0–1). No serious operation-associated complications occurred, and the median postoperative length of stay was 9 days (6–16). The most common immune-associated adverse events (AEs) were of grade 1–2: 8 patients had skin responses, 2 of which were of grade 3 or 4, and all others were of grade 1 or 2, with rash being the most common AE; 3 patients had endocrine AEs (mainly hypothroidism/hyperthroidism), and one patient had grade 4 hemorrhage, which might have been associated with apatinib. Some parameters that may be potential predictive biomarkers of immune-associated AEs in clinical practice, especially when immune checkpoint inhibitors are combined with chemotherapy and or targeted drugs^21^.

Sintilimab in combination with doublet chemotherapy and apatinib might offer an opportunity to cure stage IV gastric cancer.
